# MicroRNAs as Potential Tools for Predicting Cancer Patients’ Susceptibility to SARS-CoV-2 Infection and Vaccination Response

**DOI:** 10.3390/cells11152279

**Published:** 2022-07-23

**Authors:** Tânia R. Dias, Francisca Dias, Ana Luísa Teixeira, Hugo Sousa, Júlio Oliveira, Rui Medeiros

**Affiliations:** 1 Molecular Oncology and Viral Pathology Group, Research Center of IPO Porto (CI-IPOP) & RISE@CI-IPOP (Health Research Network), Portuguese Oncology Institute of Porto (IPO Porto)/Porto Comprehensive Cancer Center (Porto.CCC), 4200-072 Porto, Portugal; tania.dias@ipoporto.min-saude.pt (T.R.D.); francisca.carvalho.dias@ipoporto.min-saude.pt (F.D.); ana.luisa.teixeira@ipoporto.min-saude.pt (A.L.T.); hugo.sousa@ipoporto.min-saude.pt (H.S.); 2 Laboratory Medicine, Clinical Pathology Department, Portuguese Oncology Institute of Porto (IPOPorto)/Porto Comprehensive Cancer Center (Porto.CCC), 4200-072 Porto, Portugal; 3 Department of Oncology, Portuguese Oncology Institute of Porto (IPO Porto)/Porto Comprehensive Cancer Center (Porto.CCC), 4200-072 Porto, Portugal; julio.oliveira@ipoporto.min-saude.pt; 4 Biomedicine Research Center (CEBIMED), Research Inovation and Development Institute (FP-I3ID), Faculty of Health Sciences, Fernando Pessoa University (UFP), 4249-004 Porto, Portugal; 5 Abel Salazar Institute for the Biomedical Sciences (ICBAS), University of Porto, 4050-513 Porto, Portugal; 6 Research Department, Portuguese League Against Cancer Northern Branch (LPCC-NRN), 4200-172 Porto, Portugal

**Keywords:** COVID-19, vaccine, miRNAs, immune response, cancer

## Abstract

Coronavirus disease (COVID-19) is an infectious disease that is caused by a highly contagious and severe acute respiratory syndrome—coronavirus 2 (SARS-CoV-2). This infection started to spread across the world in 2019 and rapidly turned into a global pandemic, causing an urgent necessity for treatment strategies development. The mRNA vaccines against SARS-CoV-2 can trigger an immune response, providing genetic information that allows the production of spike glycoproteins. MiRNAs play a crucial role in diverse key cellular processes, including antiviral defense. Several miRNAs are described as key factors in SARS-CoV-2 human infection through the regulation of ACE2 levels and by the inhibition of SARS-CoV-2 replication and spike expression. Consequently, these molecules have been considered as highly promising biomarkers. In numerous human malignancies, it has been recognized that miRNAs expression is dysregulated. Since miRNAs can target SARS-CoV-2-associated mRNAs, in cancer patients, the deregulation of these molecules can impair the immune response to the vaccines. Therefore, in this review, we propose a miRNA profile of seven SARS-CoV-2-related miRNAs, namely miR-214, miR-98-5p, miR-7-5p, miR-24-3p, miR-145-5p, miR-223-3p and miR-15b-5p, that are deregulated in a high number of cancers and have the potential to be used as prognostic biomarkers to stratify cancer patients.

## 1. Introduction

Coronavirus disease 2019 (COVID-19) is the clinical manifestation of severe acute respiratory syndrome coronavirus 2 (SARS-CoV-2) [1]. This infection started to spread across the world in 2019, increasing hospitalization rates in several countries, and rapidly turned into a global pandemic [2]. Considering the high infection rate, which can affect an elevated percentage of individuals in each community in a short period, the mortality rate and the death risk estimation are related to the breakdown of the healthcare systems [3,4]. Therefore, on March 2020, the World Health Organization (WHO) declared a pandemic, leading to the emergence of public health strategies to contain the outbreak, including social confinement and movement restriction [2,5]. Reported illnesses have ranged from very mild (including some with no reported symptoms) to severe, including illness resulting in death [4]. Despite the worldwide spread, the clinical and epidemiological patterns of COVID-19 remain unclear [6]. The first epidemiological and clinical investigations showed that most COVID-19 cases were attributed to elder or middle- aged men, with a mean incubation period of 5.2 days [7,8]. Aside from age and gender, there are also pre-existing conditions that are considered to be risk factors for SARS-CoV-2 infection, namely: hypertension, obesity, diabetes mellitus, asthma, chronic obstructive pulmonary disease, chronic kidney disease, smoking, and diseases that induce an immunosuppression state such as cancer [9].

Cancer has a major impact on society, with more than 18 million new cases per year globally [10]. Cancer patients are more susceptible to infection compared to healthy people and non-cancer patients [11,12]. This predisposition has been historically related to the systemic malignancy-related immunosuppressive state and to active disease-oriented treatments, such as chemotherapy, immunotherapy, radiotherapy, and surgery [13,14].

SARS-CoV-2 is a member of the Coronaviridae family and consists in an enveloped, single-stranded RNA virus with positive polarity and a genome of approximately 30 kilobases [15]. Like an mRNA, the virus genome consists of a 5′ cap structure together with a 3′ poly (A) tail that translates its proteins. Coronaviridae viruses contain similar genomic RNA (gRNA) compositions, including two open reading frames (ORF1a and ORF1b), which encode for the RNA-dependent RNA polymerase (RdRp) and nonstructural proteins (nsps) [16]. ORF1a contributes to the production of nsp1–nsp11, while the rest of the nsps (nsp12–nsp16) originate from ORF1b [17]. In addition to this, one-third of the genome at the 3′ end encodes for the viral structural proteins’ surface (S), envelope (E), membrane (M), and nucleocapsid (N) [17,18]. Moreover, the genomes of Coronaviridae viruses also contain multiple structurally conserved elements within the 5′ and 3′ untranslated regions (UTRs) that have been suggested to play roles in viral replication. These elements include three stem-loops (SL1, SL2 and SL3) within the 5′ UTR, as well as a bulged stem-loop (BSL), pseudoknot (PK) stem-loop, and hypervariable region (HVR) within the 3′ UTR. The spike glycoprotein of SARS-CoV-2 comprises the receptor binding domain (RBD) in the S1 subunit, which binds with the angiotensin-converting enzyme 2 (ACE2), allowing the penetration of the virus into the endothelial and epithelial cells and consequently activating infection [19,20]. The S2 subunit improves the fusion of viral and host cell membranes that is activated by the transmembrane protease serine 2 (TMPRSS2) [20]. Then, the virus affinity to infect certain cells may be related to the multiple organ distribution of ACE2, the functional receptor for SARS-CoV-2 [21]. Consequently, spike glycoprotein and its RBD are crucial targets for vaccination and therapeutic improvement [22,23].

The global pandemic has caused an urgent necessity for vaccine development promoting the emergence of mRNA vaccines against SARS-CoV-2 [23,24]. These mRNA vaccines provide genetic information in the form of mRNA allowing the production of viral proteins by the host—more specifically the spike glycoproteins—to trigger an immune response [23,25]. However, it is important to note that, in the same way that some subsets of the population are more prone to SARS-CoV-2 infection due to immunosuppression (either caused by cancer or other immune disorders), the same applies for their capacity of vaccination response. In fact, since cancer patients are not included in vaccination clinical trials, there is still a considerable uncertainty regarding the efficacy of the SARS-CoV-2 vaccines that are available, as well as the extent of the humoral and cellular immune responses and the impact of related side effects [26]. In fact, cancer patients have a three-fold higher infection risk than the general population [27]. Therefore, it is important to identify and study new potential biomarkers and therapeutic targets for this disease. Moreover, patients with lung or hematological cancers and those who receive active chemotherapy treatment are at a greater risk of SARS-CoV-2 infection, due to an increased immunosuppression state [28]. In addition to an increased susceptibility to SARS-CoV-2 infection, the immunosuppressive state of cancer patients also makes them more prone to vaccination failure [28]. However, due to the novelty of the mRNA vaccination field, the mechanisms behind vaccination responsiveness and immunity development are not fully understood. Therefore, it is important to identify and study new potential biomarkers that can predict and monitor these patients’ susceptibility to SARS-CoV-2 infection and responsiveness to vaccination.

Currently, there is a massive investment in circulating microRNAs (miRNAs) research, due to their potential use as biomarkers for innumerous conditions. The intensive research of the past few years has demonstrated that miRNAs are secreted in several of the body fluids (e.g., blood, plasma, serum, saliva, urine, etc.) that are routinely examined in patients; are stable and resistant to degradation; and are easy to quantify through molecular biology techniques such as real time PCR [29,30,31]. This set of characteristics make miRNAs excellent biomarker candidates, with the advantage that they can be obtained through non-invasive or minimally invasive methods. MicroRNAs (miRNAs) are small non-coding RNAs of 18–25 nucleotides that play a key role in the regulation of gene expression through the post-transcriptional suppression of mRNAs [32,33]. MiRNAs have been shown to regulate every aspect of cellular activity, including differentiation and development, metabolism, proliferation and apoptosis [34]. In fact, they can regulate approximately 30–70% of human gene expression [35]. These molecules can bind directly to mRNA targets by complementarity, causing their degradation or suppressing the translation process [32]. Thousands of human protein coding genes are regulated by miRNAs, reinforcing the idea that miRNAs are master regulators of diverse biological processes with an impact in the body physiological responses [34]. Over the past decade, it has been established that miRNAs expression is dysregulated in several human malignancies, consequently affecting the hallmarks of cancer and having either an oncogenic or tumour suppressor role [36].

In addition to this, miRNAs also play a central role in immunity development and host antiviral defense [1,33]. In fact, miRNAs have been considered as highly promising biomarkers that can, among several functions, regulate immunity-related gene targets through complex networks of virus–host cell interactions [1]. Several miRNAs are described as key factors in SARS-CoV-2 human infection, regulating the inflammation, interfering with the innate immune response and assuming antiviral roles [2,37]. Further, some miRNAs were reported as crucial for virus entrance in the host cells that were involved in ACE2 levels regulation [38,39]. Furthermore, there are diverse studies demonstrating that miRNAs inhibit SARS-CoV-2 replication and inhibit spike expression [40,41]. Taking this into consideration, miRNAs can hold a positive or negative role in virus-related processes in three ways: (1) direct binding to the viral genome; (2) binding to viral transcripts; (3) binding to host transcripts [42]. Thus, human miRNAs can have a dual role: they can promote the stability and replication of viral RNA, or they can reinforce the host antiviral response. Consequently, miRNAs are considered as promising tools to explore the regulatory networks behind the immune response to COVID-19 infection and to vaccination [43,44].

Therefore, the abnormal miRNAs expression known in cancer patients may also contribute to the severity of SARS-CoV-2 infection and diminish the immune response to the vaccines. Hence, the scope of this review is to gather and systematize the available information regarding the known miRNAs that are involved in SARS-CoV-2 regulation and their expression patterns in the cancer context.

## 2. Evidence Acquisition

A literature search in PubMed was conducted using the search terms “microRNA” and “SARS-CoV-2”. The literature analysis included 350 articles that were published between 2020 and 2022. The articles were manually curated and selected by the relevance of their findings, namely, the validated interaction between a miRNA and a SARS-CoV-2 related mRNA. Of the 350 articles that were found, 319 were excluded. The exclusion criteria for the collected articles were as follows: (1) non-human miRNAs; (2) no association between miRNAs and SARS-CoV-2-related mRNAs; (3) articles based only on bioinformatic predictions or in silico analysis of interactions between miRNAs and SARS-CoV-2 related mRNAs; (4) editorials, comments and protocols; (5) individual papers that were already included in meta-analysis or reviews. For each study, information was extracted concerning the following characteristics: the name of the miRNA, the mRNA target, and validation of the miRNA–mRNA interaction through robust methods.

## 3. Evidence Synthesis

**Figure 1:**
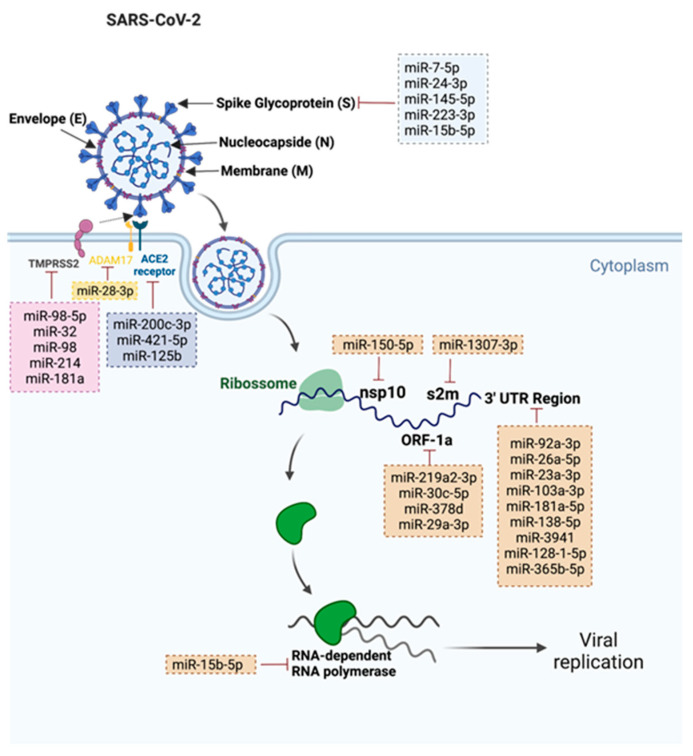
Representation of the 31 validated SARS-CoV-2 miRNAs and their respective targets. This figure was created at BioRender.com (accessed on 12 July 2022).

A total of 31 miRNAs were found. The gathered information is summarized in Figure 1. The relevant miRNAs were divided into two categories: (1) miRNAs that target SARS-CoV-2 (2) miRNAs that target SARS-CoV-2 related proteins.

### 3.1. miRNAs That Target SARS-CoV-2

We found a total of 22 human miRNAs that were involved in the targeting of several components of SARS-CoV-2, such as: 3′–untranslated regions (UTR); open reading frames (ORF); stem-loop II motif (s2m); and RNA template components of non-structural protein 10 (nsp10), spike protein, and RNA-dependent RNA polymerase (RdRp). Nine miRNAs were involved in the targeting conserved 3′–UTR of the viral genome. Park and colleagues observed that miR-92a-3p, miR-26a-5p, miR-23a-3p, miR-103a-3p and miR-181a-5p from placenta stem-cell-derived extracellular vesicles (EVs) were able to bind to 3′-UTR regions of SARS-CoV-2 and suppress RNA replication, consequently leading to the suppression of the virus-mediated pro-inflammatory response in human bronchial cells and lung fibroblasts [45]. In addition to this, Barreda-Manso and co-workers described that miR-138-5p, miR-3941, miR-128-1-5p and miR-365b-5p were also able to bind to 3´-UTR regions of SARS-CoV-2 [46]. While some of the SARS-CoV-2 3′-UTR is variable in sequence, the virus contains a highly conserved 41-nucleotide (nt) stem-loop II motif (s2m) within the terminal portion of the HVR (hypervariable region) [47]. Imperatore and colleagues highlighted the potential role of the s2m element in mediating the viral genome dimerization, suggesting its potential application as a drug target. Moreover, the authors observed that host miR-1307-3p was able to bind and inhibit s2m [47]. Akula and co-workers observed that the decline in plasma levels of miR-150-5p in COVID-19 patients could enhance SARS-CoV-2 infection [48]. In fact, these authors demonstrated that miR-150-5p was able to lower SARS-CoV-2 infection in vitro by targeting the coding strand of nsp10 and suggested that downregulation of this miRNA could be a mechanism to promote SARS-CoV-2 infection [48]. Six miRNAs were involved in the targeting of the spike (S) protein. Wang and colleagues demonstrated that miR-7-5p, miR-24-3p, miR-145-5 and miR-223-3p were able to directly target the S protein and inhibit SARS-CoV-2 replication [40]. Moreover, the authors also observed that these miRNAs were markedly decreased in elderly and diabetic patients when compared to young healthy volunteers [40]. Siniscalchi and colleagues observed that endogenously expressed lung miRNAs were able to bind and inhibit viral targets. The authors observed that miR-219a2-3p, miR-30c-5p, miR-378d and miR-29a-3p were able to bind to ORF1a, and miR-15b-5p was able to bind to spike ORF and repress plasmid-driven spike expression [49]. Moreover, these authors were also able to demonstrate that synthetic miRNA mimics of the miRNAs that were studied could be used to inhibit SARS-CoV-2, which highlights the potential of miRNAs as a therapeutic approach to fight the viral infection. In addition to spike ORF, miR-15b-5p was also described as being able to target the RNA template component of RdRp, further contributing to the suppression of viral infection and proliferation [50].

### 3.2. miRNAs That Target SARS-CoV-2 Related Proteins

We found a total of nine miRNAs involved in the targeting of key proteins that allow the entry of SARS-CoV-2 into the host cells—more specifically, TMPRSS2, ACE2 and ADAM7.

ACE2 acts as a key receptor for the spike of SARS-CoV-2, and it is crucial for the virus entry into the cells. MR-200c-3p and miR-421-5p can target ACE2, and their expression was decreased in the blood of SARS-CoV-2 patients at hospital admission, also suggesting a relation with the degree of infection [39]. On the other hand, Papannarao observed an increased expression of miR-200c in the blood of obese patients and suggested that increased angiotensin II, followed by inhibition of ACE2 through miR-200c targeting, may increase the severity of the SARS-CoV-2 infection in obese people [51]. Moreover, despite SARS-CoV-2 infection primarily manifesting as an acute respiratory illness that is accompanied by interstitial and alveolar pneumonia, it also affects multiple organs, such as the heart, digestive tract, blood, central nervous system, and the kidney. This is because ACE2 is widely expressed in the lungs, intestine, liver, testis, central nervous system, heart tissue and the kidneys [52]. In fact, in a review article, Widiasta and colleagues highlighted the importance of kidney-specific miR-125b in the modulation of ACE2 expression and COVID-19-associated nephropathy [53]. In addition to this, Xu and colleagues reported that miR-8-3p inhibits A disintegrin and metalloproteinase 17 (ADAM17)-dependent ACE2 ectodomain shedding in 293T cell treated with the S protein of SARS-CoV-2, suggesting its potential role as a therapeutic approach in the prevention and treatment of SARS-CoV-2 infection [54]. Another important protein for SARS-CoV-2 entry into the cells is TMPRSS2, which is involved in the fusion of viral and host cell membranes. Matarese and colleagues mechanistically validated miR-98-5p as a regulator of TMPRSS2 transcription in lung and umbilical vein endothelial cell types [55]. In addition to this, Kaur and co-workers silenced TMPRSS2 in Caco-2 cells with the transfection of miR-32, miR-98 and miR-214, and were able to achieve maximum gene suppression with miR-32 at a concentration of 40 nM [56]. It was also described that miR-181a, which also targets TMPRSS2, was only downregulated in severe cases of COVID19 and was associated with delayed viral clearance after infection [57].

## 4. SARS-CoV-2-Related miRNAs Deregulation Impact on Cancer Patients’ Infection Susceptibility and Vaccination Response

**Figure 2:**
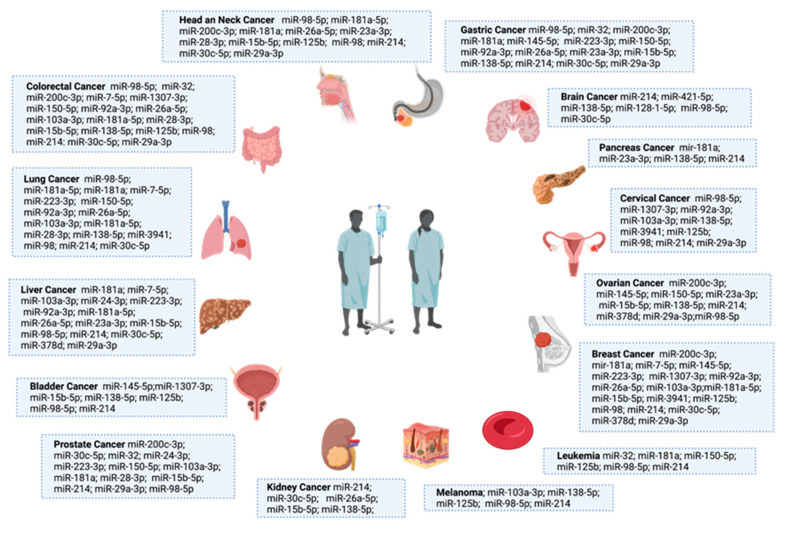
SARS-CoV-2 related miRNAs deregulated among 15 cancer types (gastric, brain, pancreas, ovarian, breast, leukemia, melanoma, kidney, prostate, bladder, liver, lung, colorectal and head and neck). This figure was created at BioRender.com (accessed on 6 May 2022).

MiRNA deregulation is well established in cancer, influencing its biogenesis and the evolution of the disease. We then endeavored to see if there were data available regarding these 31 SARS-CoV-2-related miRNAs in the cancer context—more specifically, in 15 common cancer types. The information is summarized in Figure 2. Colorectal, lung, breast and liver cancers were those that had more SARS-CoV-2-related miRNAs that were described as deregulated. Moreover, we also observed that several of those miRNAs were common to several types of cancer (Figure 2). Interestingly, of the 15 cancer types that we selected, miR-214 was reported as deregulated in all of them [58,59,60,61,62,63,64,65,66,67,68,69,70,71,72]. As previously stated, this miRNA targets TMPRSS2, which is crucial for SARS-CoV-2 entry into the cells. Therefore, a downregulation of this miRNA can lead to an increased expression of TMPRSS2 and an increased susceptibility to SARS-CoV-2 infection. In addition to this, miR-214 is known for is tumorigenic and suppressive roles in cancer, and it is also being considered as a potential biomarker and therapeutic agent [73], apart from in brain and pancreatic cancer, and melanoma were it as an oncogenic role (Figure 2) [58,59,60,61,62,63,64,65,66,67,68,69,70,71,72]. Moreover, miR-98, another miRNA that targets TMPRSS2, was reported as deregulated in 14 of the 15 cancer types that are displayed in Figure 2 (it was not described in kidney cancer). It was also downregulated in all of them except for prostate cancer, where it was described as upregulated. This information suggests that the downregulation of these miRNAs may be related to an increased susceptibility of cancers patients to develop SARS-CoV-2 infection, but this hypothesis needs to be validated.

Another important issue is the five miRNAs (miR-7-5p, miR-24-3p, miR-145-5p, miR-223-3p and miR-15b-5p) that target S protein sequences and are also deregulated in the cancer context [74,75,76,77,78,79,80,81]. This is because some of the SARS-CoV-2 vaccines are S protein mRNA fragments that are delivered to the cells through a lipid vesicle delivery approach [82]. Therefore, if the host has an overexpression of miRNAs that target S protein mRNA sequences, one can hypothesize that this host is more prone to experience reduced vaccine efficacy. In addition to other biological processes and pathways, all these miRNAs have already been reported as implicated in cancer therapy resistance or comorbidities. MiR-24-3p [74,75] and miR-7-5p [76] are associated with chemotherapy resistance; miR-145-5p [77,78] has been associated with cancer stemness and therapy resistance; miR-15b-5p [79] has been associated with doxorubicin-induced cardiotoxicity; and miR-223-3p [80,81] with radioresistance. This information suggests that the overexpression of these miRNAs in cancer patients may be related to an increased susceptibility of SARS-CoV-2 mRNA-based vaccination failure, but once again, this is a hypothesis that needs to be validated.

### Role of miR-214, miR-98-5p, miR-7-5p, miR-24-3p, miR-145-5p, miR-223-3p and miR-15b-5p in the Cytokine Storm Regulation

The cytokine storm is defined as an excessive immune response to external stimuli, such as an infection or even vaccination. The pathogenesis of the cytokine storm is complex, and the disease progresses rapidly, leading to the excessive production of cytokines by the immune system that can ultimately result in the death of the patient [83]. Recent evidence shows that, during the COVID-19 pandemic, the severe deterioration of some patients was closely related to the cytokine storm in their bodies [84]. Therefore, it is also important to study biomarkers that can predict and monitor this complex immunological phenomenon.

Based on all of the information described above, we identified miR-214, miR-98-5p, miR-7-5p, miR-24-3p, miR-145-5p, miR-223-3p and miR-15b-5p with particular interest. These miRNAs are key players in the regulation of SARS-CoV-2 human infection and are also deregulated in several types of cancer; therefore, we wished to check their role in the inflammatory process. Concerning miR-214, Sun et al. demonstrated that this miRNA could target PD-L1 to regulate the immune response of diffuse large B-cell lymphoma by modulating IL-10, IFN-γ and TNF-α expression [85]. Additionally, in ulcerative colitis, it seems that the downregulation of miR-214 may contribute to the pathogenesis of this disease. It occurs since miR-214-3p directly targets STAT6, which is upregulated in ulcerative colitis patients, and enhances the pathogenesis of this illness [86]. Regarding miR-98-5p, Wang et al. reported that this miRNA negatively modulates IL-6 expression in rheumatoid fibroblast-like synoviocytes, serving as a potential regulator of the inflammatory process. Therefore, they proposed that the manipulation of miR-98-5 could be a potential clinical intervention, serving as an inhibitor of IL-6 expression in rheumatoid arthritis [87]. On the other hand, this miRNA also targets IL-10, which is an anti-inflammatory cytokine and plays an important role in maintaining intestinal homeostasis and the mucosal barrier. Therefore, miR-98-5p may play a key role in the aggravation of ulcerative colitis, since it diminishes IL-10 expression [88]. MiR-7-5p was described as being able to inhibit melanoma growth and metastasis through the inactivation of NF-κB signaling, which leads to the decreased of NF-κB target genes expression, such as IL-1β, IL-6 and IL-8 [89]. In turn, Lin et al. demonstrated that the overexpression of miR-24-3p promoted cell proliferation, inhibited apoptosis, and increased cell migration and invasion in prostate cancer. This miRNA targets and suppresses the suppressor of cytokine signaling 6 (SOCS6), which acts as a tumor suppressor, due to the inhibition of cytokine signaling pathways [90]. Concerning miR-145-5p, Zhuang et al. showed that it was downregulated in colon cancer. Moreover, they suggested that this miRNA acts as a tumor suppressor through targeting chemokine (C-X-C motif) ligand 1 (CXCL1), which is overexpressed in colorectal cancer and facilitates metastasis and the progression of tumorigenesis [91]. MiR-223-3p can also interact with cytokines and modulate the inflammatory response. In a glioblastoma in vitro study, Ding et al. verified that treatment with miR-223-3p mimic inhibits cell proliferation and migration via decreasing numerous inflammation-associated cytokines, such as interleukin-1β, monocyte chemoattractant protein-1, IL-8 and IL-18 [91]. Furthermore, in adipose stem cells, it was confirmed that miR-223-3p directly affects the expression of the inflammatory cytokines IL-6, IL-1β and TNF-α [92]. MiR-15b-5p also interferes with the levels of some cytokines. In a high glucose-induced podocyte injury, it was shown that this miRNA was able to ameliorate the patient’s condition, attenuating the expression of IL-1β, TNF-α, and IL-6 [93]. Based on these examples, we can conclude that all of these miRNAs have the capacity to modulate the inflammation process and interfere with the immune response through the regulation of cytokine levels.

## 5. MiRNAs Applications in Cancer Patients’ Management Regarding SARS-CoV-2

**Figure 3:**
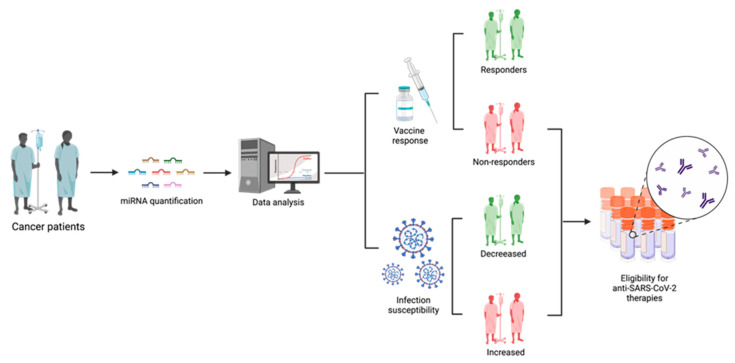
Schematic representation of the applicability of using miRNA profiles to stratify cancer patients according to their prognostic in terms of SARS-CoV-2 infection risk and vaccination responsiveness. This figure was created at BioRender.com. (accessed on 12 July 2022).

The use of circulating miRNAs as clinical biomarkers has been explored under a variety of conditions, including cancer and viral infections [94,95]. MiRNAs are considered to be robust, sensitive, and cost-effective biomarkers that can add additional information to the clinical variables that are already used in the clinical practice [58,96]. In fact, several miRNA-based diagnostic and therapeutic products are already in clinical trial phase and expected to enter the market in the next few years [97]. Therefore, the study and use of miRNA profiles to stratify cancer patients according to their risk of SARS-CoV-2 infection and vaccination effectiveness seems a promising personalized approach to improve the current management of these patients (Figure 3). Moreover, miRNAs can be obtained through minimally invasive methods, such as a blood sample, and their isolation and quantification protocols are simple and less time-consuming when compared with other type of molecules [29,30]. In fact, the implementation of a stratification algorithm would allow the selection of patients that could benefit from the emerging SARS-CoV-2 therapeutic approaches, such as the Long-Acting Antibodies (LAAB) AZD7442, which are currently in phase III clinical trials (ClinicalTrials.Gov NCT04507256) and show promising results in reducing the risk of severe COVID-19 or death. LAABs mimic natural antibodies and can block the binding of the SARS-CoV-2 virus to host cells [98]. Therefore, their potential is being tested to treat and prevent disease progression in patients that are already infected with the virus, as well as to be given as a preventative intervention prior to exposure to the virus.

## 6. Discussion

The COVID-19 pandemic tested the resilience of health-care systems worldwide in an unprecedented manner and forced clinicians to rethink and adapt the diagnostic and therapeutic approaches based on the local prevalence of the virus in their communities [28]. This includes the cancer patient population, which is particularly susceptible to SARS-CoV-2 infection and complications. Therefore, there is an urgent need for a stratification system that can allow the selection of cancer patients with an increased risk of infection and/or lower vaccination response, in order to help oncologists to prioritize patients and optimize health-care system resources to provide the best patient care, given the circumstances.

Considering that miRNAs are among the earliest molecular regulators, circulating miRNAs could be potential biomarkers in the early identification of those at the highest risk of developing COVID-19 and related complications. In fact, there are a few recent studies describing plasma miRNA profiles that are capable of predicting the ICU stay of patients and the severity of SARS-CoV-2 infection [99,100]. Moreover, other authors have proposed that miRNAs could be used as a therapeutic approach against COVID-19 [101,102]. However, there are no studies covering the applicability of using miRNA profiles to stratify cancer patients according to their risk of developing SARS-CoV-2 infection and/or decreased vaccination response. In this review, we summarized the available information regarding the miRNAs that interact with SARS-CoV-2 and SARS-CoV-2-related genes that are already validated and checked if those miRNAs were reported as deregulated in cancer. Based on this information, we propose a miRNA profile of seven SARS-CoV-2-related miRNAs (miR-214, miR-98-5p, miR-7-5p, miR-24-3p, miR-145-5p, miR-223-3p and miR-15b-5p) that are deregulated in a high number of cancers and have the potential to be used as prognostic biomarkers to stratify cancer patients. Moreover, we also gathered information regarding the impact of these miRNAs in immune-related molecules, such as cytokines and chemokines, that can also have an impact in the development of the cytokine storm. However, it is important to note that future studies require the validation of this miRNA profile in the clinical setting to validate our hypothesis before integrating it into the decision-making algorithms that are used by clinicians. Since miRNAs are stable and detectable using small quantities of human body fluids, it would be possible to conduct miRNA quantification in a high number of cancer patients in a short period of time using the samples that are already stored at the biobanks of the hospitals and cancer institutes. Overall, miRNA research has demonstrated a lot of potential in the cancer field in the last few years, ranging from biomarker potential to personalized therapeutic applications. Thus, it makes sense to expand the range of application of prognostic biomarkers to further stratify cancer patients according to their prognostic, regarding SARS-CoV-2 infection and vaccination benefit.

## Data Availability

Not applicable.
